# Proteases as Tools for Modulating the Antioxidant Activity and Functionality of the Spent Brewer’s Yeast Proteins

**DOI:** 10.3390/molecules28093763

**Published:** 2023-04-27

**Authors:** Loredana Dumitrașcu, Andreea Lanciu Dorofte, Leontina Grigore-Gurgu, Iuliana Aprodu

**Affiliations:** Faculty of Food Science and Engineering, Dunarea de Jos University of Galati, 111 Domneasca Str., 800008 Galati, Romania; loredana.dumitrascu@ugal.ro (L.D.); andreea.dorofte@ugal.ro (A.L.D.); leontina.gurgu@ugal.ro (L.G.-G.)

**Keywords:** spent brewer’s yeast, enzymatic hydrolysis, peptides, antioxidant activity, functional properties

## Abstract

The functionality of the peptides obtained through enzymatic hydrolysis of spent brewer’s yeast was investigated. Hydrolysis was carried out for 4–67 h with bromelain, neutrase and trypsin. The resulting hydrolysates were characterized in terms of physical-chemical, antioxidant and techno-functional properties. The solid residues and soluble protein contents increased with the hydrolysis time, the highest values being measured in samples hydrolyzed with neutrase. Regardless of the hydrolysis time, the maximum degree of hydrolysis was measured in the sample hydrolyzed with neutrase, while the lowest was in the sample hydrolyzed with trypsin. The protein hydrolysate obtained with neutrase exhibited the highest DPPH radical scavenging activity (116.9 ± 2.9 μM TE/g dw), followed by the sample hydrolyzed with trypsin (102.8 ± 2.7 μM TE/g dw). Upon ultrafiltration, the fraction of low molecular weight peptides (<3 kDa) released by bromelain presented the highest antioxidant activity (50.06 ± 0.39 μM TE/g dw). The enzymes influenced the foaming properties and the emulsions-forming ability of the hydrolysates. The trypsin ensured the obtaining of proteins hydrolysate with the highest foam overrun and stability. The emulsions based on hydrolysates obtained with neutrase exhibited the highest viscosity at a shear rate over 10 s^−1^. These results indicate that the investigated proteases are suitable for modulating the overall functionality of the yeast proteins.

## 1. Introduction

The global production of beer reached about 1.86 billion hL in 2021, out of which around 342 million hL were produced only in Europe [1,2]. The brewing industry generates about 0.4 billion tons of spent brewer’s yeast (SY) each year and even more billion tons of spent grain and hops [3]. Despite their high circular economy potential, either in food, feed or for pharmaceutical and cosmetic purposes, most of the time SY is used as raw material for animal feed or discarded as waste [3]. SY has a very appealing composition as, based on dry weight, it contains high-quality proteins (45–60%), carbohydrates (15–35%), nucleic acids (4–8%), lipids (4–7%), B vitamins complex, minerals and dietary fibers [4,5,6]. The SY proteins contain essential amino acids in quantities that meet the FAO/WHO/UNU reference profile [7,8]. In addition, SY represents an excellent source of bioactive peptides, making it very attractive for various applications, such as for developing nutraceuticals and functional foods.

Moreover, as the global population is expected to reach about 10 billion by 2050, doubling the food demand in the next decades, the valorization of different by-products such as SY represents a sustainable, economical and healthy alternative for acquiring food-grade proteins for the increasing population [9]. Compared to other alternative food protein sources, SY has the Generally Recognized as Safe status, being available throughout the year. The initial step in the recovery of proteins and generation of peptides from SY consists in disrupting the cell walls to release the intracellular components. The disruption of the cell walls and further protein release can be achieved by using individual or various combinations of physical, chemical or enzymatic treatments. Enzymatic hydrolysis is one of the most frequently applied methods for the production of protein hydrolysates and peptides, being considered efficient, safe and relatively cheap compared to other methods [10]. However, the characteristics of the peptides usually depend on the protein chain from which they were released and the type of enzyme used for hydrolysis [11]. As reviewed by Oliveira et al. [6], endopeptidases such as Alcalase^®^, Corolase^®^, Papain^®^ and Brauzyn^®^, alone or in admixture with exopeptidases (Flavourzyme^®^ and Promod^®^) and other proteases (Protamex^®^), are most frequently used for generating protein/peptide rich extracts from SY. Many in vivo and in vitro studies performed on SY hydrolysates or isolated peptides have demonstrated antioxidant, immunomodulatory, antihyperglycemic, antihypertensive, antithrombotic, lipid-lowering, antimicrobial, antiulcer, and antiproliferative activities [12]. In particular, high antioxidant activity values of the SY extracts were reported by Podpora et al. [13], who performed autolysis assisted by enzymatic hydrolysis with papain, and Marson et al. [5], who employed enzymatic hydrolysis with Brauzyn^®^, Protamex^®^ and Alcalase^®^.

The objective of this study was to investigate the impact of using three different proteases, namely bromelain, neutrase and trypsin, for SY proteins lysis on the antioxidant activity and technological functionality of the resulting hydrolysates. Although the potential of these enzymes for obtaining hydrolysates with increased bioactivity was demonstrated on several protein sources of animal or vegetal origin [14], there are no studies that focus on the functionality of the peptide-rich hydrolysates released by bromelain, neutrase and trypsin from the SY proteins.

## 2. Results and Discussion

### 2.1. The Effect of Enzymatic Hydrolysis on the Recovery of SRC and SPC

Hydrolysis with exogenous proteolytic enzymes represents an efficient method of cell lysis. Depending on the amount they are added, these enzymes together with endogenous enzymes accelerate leakage and recovery of intracellular compounds [6]. In the present study, the recovery of cellular compounds resulting from cell wall breakdown was measured in terms of SRC (Table 1) and SPC (Table 2). The SRC and SPC were influenced by the hydrolysis time that had a positive effect on the recovery of intracellular components. The maximum recovery of the solids from the yeast cells was obtained upon 21 h of hydrolysis, while further increase in the hydrolysis had no significant effect on the recovery of soluble solids. The type of enzyme also had a significant effect on the SRC and SPC. Compared to the control sample, after 4 h of hydrolysis, the SRC increased by 29.1%, 73.7% and 50% in the BSY, NSY and TSY samples, respectively. Among all tested enzymes, the hydrolysates produced with neutrase contained the highest SRC, regardless of the hydrolysis time. Similar results were reported in the literature, where SRC after enzymatic hydrolysis had a range of 41–61%. For example, Takalloo et al. [15] tested several extraction techniques such as autolysis, plasmolysis and enzymatic hydrolysis for the recovery of intracellular components from *Saccharomyces cerevisiae*. The authors reported that enzymatic hydrolysis resulted in the highest release of soluble solids and proteins after 48 h of hydrolysis with alcalase, when the soluble solid content increased to 52.1%. In our study, similar results were measured after 21 h of hydrolysis performed with bromelain and trypsin.

The SPC release upon SY hydrolysis varied with the enzyme and the duration of the treatment (Table 2). After 4 h of hydrolysis, the SPC of the BSY, NSY and TSY samples was 17%, 41% and 19% higher compared to the control sample. Our findings are in agreement with Marson et al. [5], who used a mixture of proteases for the 2 h hydrolysis of the spent yeast suspension and reported an increase of the protein content recovered in the extract with 16% compared to the control. Moreover, it can be seen that after 4 h of hydrolysis with neutrase the SPC was at least 1.5–1.6 times higher than in the case of bromelain and trypsin. On the other hand, it can be observed that SPC reached a plateau after 21 h of hydrolysis, regardless of the tested enzyme. Overall, regardless of hydrolysis time, the maximum SPC was obtained in the NSY sample, indicating that protein recovery was more responsive to neutrase than bromelain and trypsin. Thamnarathip et al. [16] reported a protein recovery yield on a dry basis of 31.9 ± 0.2 % for the rice bran protein samples hydrolyzed with neutrase for 8 h.

### 2.2. Degree of Hydrolysis

DH represents an essential parameter to indicate the level of hydrolysis of proteins for obtaining peptides of different sizes and amino acid sequences [17]. The DH results obtained after yeast proteins’ hydrolysis with the three different enzymes considered in the study are presented in Table 3.

After 4 h of hydrolysis, the DH reached significantly higher values for the samples where hydrolysis was assisted by exogenous enzymes: 11.50% in the BSY sample, 22.70% in the NSY sample and 12.52% in the TSY sample. The evolution of the DH over the 67 h of hydrolysis depended on the exogenous enzyme used for preparing the sample. For example, in the case of the BSY sample, the DH was 22.55% after 67 h of hydrolysis, almost double with respect to the value registered after 4 h; in TSY, the DH increased by about 50%, whereas in the sample hydrolyzed with neutrase, the DH increased only by about 19% (Table 3). In our study, the DH registered for hydrolysates obtained with neutrase was higher compared to the results of Thamnarathip et al. [16], who used neutrase to obtain bran protein hydrolysates and reported a DH of 8.34% after 6 h of hydrolysis. Xu et al. [18] used neutrase for hydrolyzing the casein, and after 12 h of hydrolysis, the DH was about 15%. From Table 3 it can be seen that, although during the first 4 h of hydrolysis, the DH was similar in TSY and BSY samples, until the end of the investigated hydrolysis time, bromelain was able to recognize and hydrolyze more peptide bonds than trypsin. These results suggest a better exposure of the peptide bonds cleaved by bromelain. Among all tested enzymes, and independent of the hydrolysis time, the highest DH was measured in the NSY sample and the lowest in the TSY sample, suggesting that the number of available cleavage sites for neutrase was higher than other endoproteases tested in this study. Trypsin is one of the most used enzymes for the production of bioactive peptides. Trypsin is able to recognize and cleave the peptide bonds involved at the C-terminal side, the positively charged amino acids lysine and arginine. In any case, the enzyme affinity towards these peptide bonds is reduced by the presence of acidic amino acids on either side of the cleavage site. Moreover, the presence of proline residue on the C-terminal side of the cleavage site will hinder the hydrolysis [19]. The DH reported in our study for yeast samples hydrolyzed with trypsin is similar to the results reported by Mirzaei et al. [20], where the sonication-trypsin hydrolysis of yeast suspensions resulted in a DH of 17.81% after 5 h at 37 °C. Most of the studies reporting on the hydrolysis of spent brewer yeast used Alcalase, Protamex™, Brauzyn^®^ and Flavourzyme™. After 2 h of spent brewer’s yeast hydrolysis with different enzymes (having the same enzymatic activity), Marson et al. [5] reported a DH ranging between 8.2 and 33.1%, the maximum DH being obtained through combining Protamex™ and Brauzyn^®^. On the other hand, the potential of using other enzymes for hydrolyzing spent brewer’s yeasts has been reported in recent studies. For example, Amorin et al. [21] used autolysis (70 °C, 5 h) followed by enzymatic hydrolysis of the spent brewer’s yeast using an extract of *Cynara cardunculus* and showed that the DH increased with time and enzyme concentration. The highest DH (about 30%) was reported after 4.5 h of hydrolysis with 4% *Cynara cardunculus* extract.

### 2.3. SDS-PAGE Analysis

**Figure 1:**
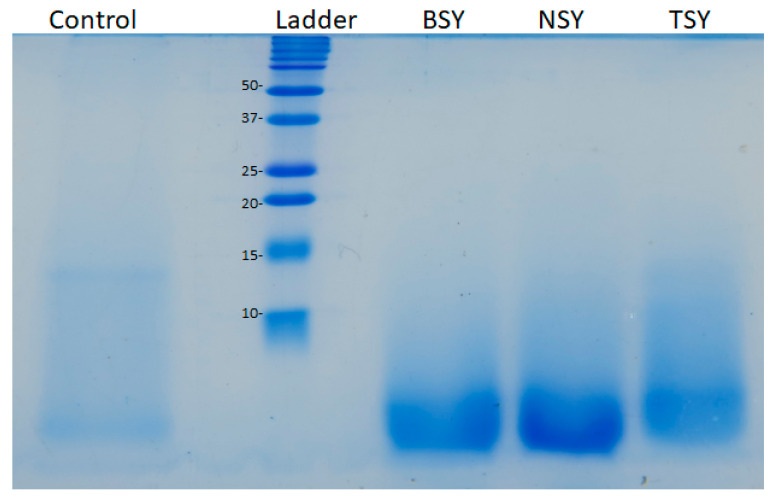
The SDS-PAGE profile of the spent brewer’s yeast hydrolyzed for 67 h, with different exogenous enzymes, and passed through ultrafiltration membranes with the cut-off of 30 kDa. The control sample in lane 1, Ladder—Dual Xtra Standard (Bio-Rad, Hercules, CA, USA), BSY—the sample treated with bromelain, NSY—the sample treated with neutrase, and TSY—the sample treated with trypsin.

The SDS-PAGE electrophoresis was conducted under reducing conditions to show that the distribution of the peptides’ molecular weight in the yeast protein hydrolysates passed through ultrafiltration membranes with a cut-off of 30 kDa. As depicted in Figure 1, the hydrolysis with the three enzymes induced the formation of small peptides, most of them having a molecular weight lower than 5 kDa. In terms of band intensity, the pattern of the samples treated with neutrase (NSY line) gave a more intense spectrum compared with those hydrolyzed with bromelain (BSY line) and trypsin (TSY line). These observations are in good agreement with the DH results showing that, after 67 h, neutrase was the most aggressive in breaking down the peptide bonds within the yeast proteins’ substrate (DH of 26.91%), followed by bromelain (DH of 22.55%) and trypsin (DH of 18.05%). Comparing the band intensity in Figure 1, one can observe the presence of higher amounts of peptides with molecular weights lower than 10 kDa in the BSY, NSY and TSY samples compared to the control obtained with no exogenous enzyme addition. The findings of the proteins’ electrophoresis can be related to the results of the soluble proteins quantified in the yeast extracts as well. After 67 h hydrolysis, the highest soluble protein content was found in the NSY, followed by BSY, TSY and the control. The same trend can be observed in the electrophoresis results, suggesting the presence of the highest amounts of low molecular weight peptides in the NSY sample. A low-intensity smear pattern was observed in the gel for all hydrolyzed samples and was associated with peptides having a molecular weight lower than 10 kDa (Figure 1).

### 2.4. Antioxidant Activity

The potential antioxidant activity of the SY hydrolysates was measured based on ABTS^+^ and DPPH scavenging-based assays, the results being presented in Table 4. The ABTS^+^ scavenging activity is based on the reduction of cation-radical ABTS^+^ into the colorless ABTS by the electron transfer of an antioxidant, while DPPH is reduced by the hydrogen atom donation of an antioxidant, changing the color of the final product from pinkish to pale yellow [22].

The results obtained using ABTS method (Table 4) showed that, after 4 h of hydrolysis, the antioxidant activity was about 11% higher in the BSY and TSY samples, and by about 13% in the NSY sample, compared to the control. Increasing the hydrolysis time generated hydrolysates with higher antioxidant activity. For all tested enzymes, a gradual increase in antioxidant activity was observed over the entire test hydrolysis time. Thus, after 67 h of hydrolysis, the antioxidant activity of the BSY and NSY samples increased by about 94% and that of the TSY sample by about 82%. The antioxidant activity was also affected by the exogenous enzyme used for hydrolysis, the highest antioxidant activity, based on the ABTS^+^ method, being calculated for NSY samples regardless of hydrolysis time, followed closely by samples hydrolyzed with bromelain and trypsin. Our results are in agreement with those reported by Vieira et al. [23]. These authors obtained protein hydrolysates from spent brewer grains by using several enzymatic techniques, and the sample hydrolyzed with neutrase, after ultrafiltration, showed the highest antioxidant potential.

Analyzing the results presented in Table 4, one can see that the antioxidant activity quantified using the DPPH method is always lower than values determined with the ABTS^+^ method. These differences might be explained by the fact that the DPPH radical presents a higher stability compared to ABTS^+^ [24] and by the differences in the reaction mechanisms standings behind the two methods. After the first 4 h of hydrolysis (Table 4), the antioxidant activity of the BSY and control samples was similar, whereas the antioxidant activity of the NSY and TSY samples was higher by 81.5% and 46.35% compared to the control. Regardless of the sample, a smaller increase of the antioxidant activity measured by the DPPH method compared to the ABTS^+^ assay was observed with increasing the hydrolysis time. The antioxidant activity measured by the DPPH method reached a maximum after 43 h of hydrolysis. Among all tested enzymes the highest antioxidant activity was measured in the NSY sample (116.9 ± 2.9 μM TE/g dw), followed by TSY (102.8 ± 2.7 μM TE/g dw), and the lowest for the BSY sample (96.51 ± 2.57 μM TE/g dw). When compared to the control sample, the maximum increase of the antioxidant activity of about 40% was measured in the sample where hydrolysis was performed with neutrase. The presence of hydrophobic amino acids in the peptide sequences exhibits an important role in the antioxidant activity [20]. Further increase of the hydrolysis time to 67 h caused the decrease of the DPPH activity of all tested samples. The DPPH scavenging activity decrease might be attributed to the advanced hydrolysis that might affect the structure and bioactivity of the active peptides [16]. Similar results have been reported in other studies, where yeast hydrolysates showed higher antioxidant activity using the ABTS^+^ method and lower for the DPPH [20,22,25].

In order to find out more information on the peptides contributing to the antioxidant activity, the samples obtained after 67 h of hydrolysis were subjected to ultrafiltration through membranes with different cut-offs, and the antioxidant activity of each permeate fraction was assessed. From Table 5 it can be seen that all permeate fractions were able to reduce the cation radical ABTS^+^. In the case of the permeates with peptides having molecular weights lower than 30 kDa, the highest ABTS^+^ scavenging activity was measured for the BSY sample (1223 ± 10 μM TE/g dw), followed closely by NSY (1203 ± 4 μMl TE/g dw) and TSY (1086 ± 23 μM TE/g dw). On the other hand, for the same fraction, when antioxidant activity was evaluated by the DPPH method, the strongest scavenging activity was measured in the TSY sample (71.08 ± 4.22 μM TE/g dw) and the lowest in the BSY sample (60.15 ± 7.53 μM TE/g dw).

Regarding permeates with peptides having molecular weights lower than 10 kDa, the highest antioxidant activity based on ABTS^+^ scavenging activity was recorded for NSY (1286 ± 10 μM TE/g dw), and for BSY (85.10 ± 5.3 μM TE/g dw) when using the DPPH method. Other authors reported that increased antioxidant potential of the 10 kDa fraction resulted after the hydrolysis with neutrase [23]. Moreover, the fraction concentrating peptides with molecular weight lower than 10 kDa exerted a protective effect against free-radical-induced cytotoxicity in Caco-2 and HepG2 cell lines.

When analyzing fractions concentrating the lowest size peptides (molecular weight < 3 kDa), one can see that the strongest scavenging activity by using ABTS^+^ assay was obtained for samples hydrolyzed with neutrase and bromelain (Table 5). However, when using the DPPH assay, the highest antioxidant activity was measured in the sample hydrolyzed with bromelain (50.06 ± 0.39 μM TE/g dw) and the lowest in the sample hydrolyzed with neutrase (27.63 ± 0.30 μM TE/g dw). Bromelain shows preferences for peptide bonds involving glutamic acid, aspartic acid, lysine or arginine residues at the N-terminal, cleaving particularly the peptide chain at arginine–alanine and alanine–glutamic acid bonds. Therefore, one can assume that in the <3 kDa fraction, these amino acids are present in higher amounts, contributing to the increased antioxidant activity of this fraction [26].

Recent studies showed the potential of using bromelain for obtaining hydrolysates with antioxidant and antimicrobial activity. Selamassakul et al. [26] showed that hydrolysis of brown rice protein with bromelain produced low molecular weight peptides that could be used to enhance the biological activity of foods, whereas Ghanbari et al. [17] found that the 7 h hydrolysis of sea cucumber with bromelain generated peptides with antimicrobial activity. Mirzaei et al. [20] used trypsin to obtain hydrolysates from yeasts, and the fraction including peptides with molecular weight < 3 kDa presented ACE inhibitory activities as well as strong DPPH and ABTS^+^ scavenging activities of 179.24 ± 4.8 μM TE/mg protein and 4653.36 ± 5 μM TE/mg protein, respectively. The results were attributed to the total content of hydrophobic amino acids with aromatic or branched side chains at each of the C-terminal tripeptide positions.

Analyzing the results presented in Table 4 and Table 5, it can be seen that, after separation through membranes with various cut-offs, the antioxidant activity of the resulting fractions was lower compared to the initial hydrolysates. Our observation is in agreement with Mirzaei et al. [20], who indicated that the overall antioxidant activity of the peptides released from *Saccharomyces cerevisiae* proteins is the result of combined actions of fractions with molecular weights < 3 kDa and 5–10 kDa.

### 2.5. Color Coordinates

The color coordinates measured on the hydrolyzed yeast slurries after the thermal inactivation of the enzymes are presented in Table 6. In the case of the control sample, the hydrolysis time had no significant effect on the luminosity coordinate. On the other hand, regardless of the exogenous enzyme used for hydrolysis, L* decreased with increasing hydrolysis duration (*p* < 0.01). The highest decrease of L* was measured in the NSY sample, followed by the BSY sample, indicating a darker color of the hydrolysates produced with neutrase and bromelain (Table 6). Moreover, similar to the results reported by Marson et al. [5], in our study, L* was negatively correlated with the soluble protein content (BSY − R2 = 0.84; NSY − R2 = 0.91; TSY − R2 = 0.94; *p* < 0.01), an indication that darker samples contain higher protein levels. The a* coordinate was influenced by both the exogenous enzyme and hydrolysis time (Table 6). For the control, BSY and NSY samples, a* coordinate increased with increasing hydrolysis time, indicating the tendency towards the red of these samples. For the TSY sample, a* coordinate decreased from 5.83 ± 0.05 to 4.98 ± 0.03, with an increasing time of hydrolysis. After 4 h of hydrolysis, the highest b* coordinate was measured for NSY and the lowest for the TSY sample (Table 6). On the other hand, after 67 h of hydrolysis, the highest increase was measured for the control sample. From Table 6 it can be seen that similar to a* values, b* values for the TSY sample increased after 8 h of hydrolysis, reaching a maximum of 12.94 ± 0.02. Similar results were reported by Bertolo et al. [27]. For control yeast suspension, the authors reported a b* value of 16.42 ± 0.02. Based on the above-mentioned results, it can be concluded that the enzymes used in this study for the yeast proteins’ hydrolysis exerted a significant effect on the color of the hydrolysates.

### 2.6. Technological Functionality of the Yeast Protein Hydrolysates

The influence of the exogenous enzyme-assisted hydrolysis on the technological functionality of the yeast proteins was determined by assessing the foaming properties and rheological behavior of the emulsions.

The foaming ability of the yeast protein hydrolysates was determined upon incorporating air into the samples at three different homogenization speeds, and the results are presented in Table 7. The foaming capacity of all samples increased with the homogenization speed. Regardless of the homogenization speed used for obtaining the foams, the samples prepared with exogenous enzymes exhibited significantly higher foaming capacity compared to the control (*p* < 0.01). As one can see in Figure 1, the exogenous enzyme-assisted hydrolysis released higher amounts of peptides with low molecular weights, contributing to the foaming capacity of the samples. As indicated by Liang et al. [28], who studied the effect of the controlled pepsin-assisted hydrolysis on the foaming properties of soy proteins, the significantly higher foaming ability of the hydrolysates might be due to the presence of the low molecular weight peptides with amphiphilic properties and more flexible structure, which are absorbed faster at the gas-water interface. The enzyme-assisted hydrolysis allows better exposure of the proteins’ hydrophobic patches which establish contacts with the gas phase, while the hydrophilic groups tend to interact with the liquid phase [29]. The lower foaming capacity of the control sample might be associated with the lower degree of hydrolysis (Table 1), which explains the higher abundance of the large molecular weight proteins and aggregates in the dispersion subjected to foaming, therefore resulting in slower diffusion to the gas-water interface. The highest overrun values were observed in the case of yeast proteins hydrolysate prepared with trypsin (FC of 85.0–132.5%). Among the enzyme-assisted hydrolyzed samples, NSY exhibited the lowest foaming ability, most probably as the result of the higher DH value (Table 3), resulting in higher amounts of peptides with very low molecular weights. A previous study of Van der Ven et al. [30], dealing with whey protein hydrolysates, indicated that the peptide fractions with 3–5 kDa have better foaming properties compared with the fraction having larger (over 20 kDa) or very small (<3 kDa) peptides.

The foam stability over 30 min of storage at room temperature ranged between 56.7 and 98.0% in case of the samples prepared with exogenous enzymes, which is significantly higher compared to the control (FS of 46.4–58.0%). The interactions established between the peptides released through enzyme hydrolysis appear to contribute to the formation of a cohesive and flexible film around the gas bubbles [29]. The higher foam destabilization tendency and liquid drainage registered for the control sample are due to the poor viscoelastic and mechanical properties of the film, which is prone to easy rupture, leading to larger air bubbles and coalescence phenomenon [28]. The homogenization speed exerted no significant influence on the foam stability (Table 7). The only exception concerns the NSY sample, which exhibited significantly lower FS when foaming at a lower homogenization speed (*p* < 0.01).

The ability of the yeast protein hydrolysates to form viscoelastic layers on the surface of the oil droplets was further assessed by determining the rheological behavior of the emulsions prepared with a volume fraction of sunflower oil of 50%. The rheological measurements on the emulsions under flow conditions indicated the shear stress increase over the entire shear rate domain considered in the study. The apparent viscosity of the emulsions measured at particular shear rate values are presented in Table 8. Regardless of the protein hydrolysate used to prepare the emulsion, the viscosity values varied depending on the applied shear rate. The highest apparent viscosity values were measured at lower shear rates for the emulsions based on control and BSY hydrolysates. On the other hand, at high shear rates no important differences in terms of the apparent viscosity were registered among the investigated emulsion samples (Table 8).

Rheological measurements under low amplitude oscillatory conditions, during the strain sweep test at a constant frequency of 1 Hz were first conducted to assess the LVR of the emulsions. The critical strain (γc) values which mark the limit of the LVR, beyond which the emulsions no longer exhibit linear viscoelastic behavior, are presented in Table 8. Similar upper limits of the LVR of 0.5–1% were previously reported by Vasilean et al. [31] for the soy protein emulsions prepared with sunflower, canola and palm oils.

The oscillatory tests based on the progressive increase of the deformation (%) also allowed the identification of the emulsion flow threshold, corresponding to the point where the phase inversion occurs, and the flowing process is considered initiated [32]. The yield strain values (γy) were recorded when the G″ values, corresponding to the viscous component of the sample, exceed the G′ values corresponding to the elastic component. The emulsions exhibited different responses to the applied strain. Except for the emulsion prepared with trypsin-assisted hydrolyzed extract, all samples exhibited a solid-like behavior, with G′ prevailing over the G″, up to strain values depending on the enzyme. The highest yield strain value of 25.22% was registered in the case of emulsion prepared with BSY, whereas the lowest (γy of 1.00%) was in the case of the NSY-based emulsion. The emulsions prepared with yeast protein hydrolysates obtained with trypsin presented higher values of G″ compared to G′ throughout the entire scanned deformation range.

**Figure 2:**
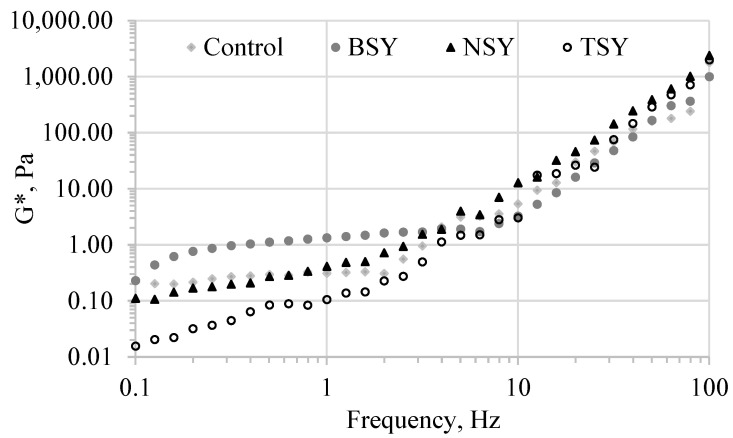
Evolution of the complex modulus (G*) during the frequency sweep tests on the emulsions prepared with an extract obtained from spent brewer’s yeast hydrolyzed for 67 h with bromelain (BSY), neutrase (NSY) and trypsin (TSY).

Frequency sweep tests were further run within the LVR at strain values below the critical strain determined for each tested emulsion (Table 8). The results of the frequency sweep test, in terms of the evolution of the complex modulus (G*), are presented in Figure 2.

The complex modulus provides information on the overall resistance to the deformation of the tested emulsions, integrating both the recoverable (elastic) and the non-recoverable (viscous) component. The values of the complex modulus represent a direct measure of the rigidity of the analyzed samples when exposed to stress below the yield stress. At low-frequency values, which provide information on the slow-motion flow behavior on a long time scale, the emulsions prepared with the yeast extract hydrolyzed with bromelain exhibited the highest G′ and G″ values and, consequently, the highest complex modulus (Figure 2), suggesting that the network structure of the emulsion is stronger compared to the other tested samples. The lowest complex modulus values were registered for the TSY-based emulsions, which exhibited liquid-like behavior, being weakly flocculated. At high-frequency values, showing the fast motion behavior at short timescales, the highest complex modulus values were registered for the TSY- and NSY-based emulsions.

## 3. Materials and Methods

Dried SY with a protein concentration of 41.96% was kindly provided by a beer factory from Ploiesti, Romania. Bromelain was provided by Carl Roth (Karlsruhe, Germany), Neutrase 5.0 BG by Novo Nordisk (Bagsværd, Denmark) and trypsin by Merck (Darmstadt, Germany), whereas o-phthaldialdehyde (OPA), N-acetyl-cysteine (NAC), sodium dodecyl sulfate (SDS), 2,2-diphenyl-1-picryl-hydrazyl (DPPH) and 2,2′-azinobis(3-ethyl-benzothiazoline-6-sulphonate) (ABTS) were purchased from Sigma Aldrich (Burlington, MA, USA). All other reagents were of analytical grade.

### 3.1. Enzymatic Hydrolysis of SY Suspensions

Dried SY was suspended in distilled water to achieve a solid concentration of 12% (*w*/*w*) and homogenized at 15,000 rpm for 10 min (Ultra Turrax^®^ IKA T18 basic and S18N-19G dispersing tool, IKA-Werke GmbH and Co. KG, Staufen, Germany). The resulting suspension with a pH of 6.5 was pretreated for 1 h at 75 °C under continuous stirring at 150 rpm. The suspension was then cooled to 50 °C and the pH was adjusted to 7.0. The concentration of the enzymes added to the yeast slurry, with respect to the solid content of the suspension, was as follows: bromelain 0.5% (BSY), neutrase 1% (NSY) and trypsin 1% (TSY). The hydrolysis was carried out for 67 h at 55 °C, while continuously shaking the samples at 100 rpm. The control sample was prepared with no enzyme addition. At the end of the hydrolysis step, the pH was adjusted to 7.0 and the enzymes were inactivated by heating the hydrolysates at 95 °C for 5 min. Cell debris was removed upon separation as residue through centrifugation at 14,000 rpm for 10 min. The liquid extract, which retained all soluble peptide fractions, was further freeze-dried (CHRIST Alpha 1–4 LD plus, Osterode am Harz, Germany).

### 3.2. Separation of Peptide from SY Hydrolysates

The supernatant resulting after centrifugation was passed through ultrafiltration membranes in an Amicon^®^ stirred cell model (Merck KGaA, Darmstadt, Germany) with a cut-off of 30 kDa, 10 kDa and 3 kDa, respectively. The antioxidant activity of the resulting supernatant was measured by using DPPH and ABTS^+^ assay.

### 3.3. Characterization of SY Hydrolysates

#### 3.3.1. Soluble Residue Content (SRC) and Soluble Protein Content (SPC)

The SRC was measured on the supernatant fraction by heating the samples in a Memmert UNB 400 oven (Memmert GmbH+Co. Kg, Schwabach, Germany) to constant weight [33]. The SPC was measured on the supernatant fraction by using the Lowry method, as previously reported by Dumitrascu et al. [33].

#### 3.3.2. Degree of Hydrolysis (DH)

The evaluation of the degree of hydrolysis was performed according to OPA spectrophotometric assay, as described by Spellman et al. [34]. Fresh OPA reagent (100 mL) was obtained by mixing 10 mL of 50 mM OPA in methanol, 10 mL of 50 mM NAC, 5 mL of SDS (20 %, *w*/*v*) and 75 mL of 0.1 M borate buffer (pH = 9.5). The reaction was initiated by mixing 400 μL of samples with 3 mL of OPA reagent, and after 15 min the mixture absorbance was measured at 340 nm (A_sample_). A blank sample was prepared by replacing the sample with distilled water. The standard sample was obtained by adding 400 μL serine standard to 3 mL of OPA solution and measuring the absorbance at 340 nm (A_standard_). The DH was calculated as indicated by Nielsen et al. [35] (Equations (1)–(3)):

DH (%) = h/h_tot_·100 (1)

where h represents the number of hydrolyzed bonds, and h_tot_ = 7.5 is the total number of peptide bonds per protein equivalent.

h = (serine-NH_2_ − β)/α (2)

serine-NH_2_ = (A_sample_ − A_blank_)/(A_standard_ − A_blank_)·0.9516 meq/L·d 100/X·P (3)

where, β was considered 0.4, α was considered 1, d is the dilution, X is the grams of the sample and P is the protein % in the sample.

#### 3.3.3. The Proteins Gel Electrophoresis

The peptides obtained upon ultra-filtration through the membrane with a cut-off of 30 kDa were analyzed by sodium dodecyl sulfate−polyacrylamide gel electrophoresis (SDS-PAGE). The concentration of the polyacrylamide was 15% in the case of the resolving gel (pH 8.8) and 4.5% in the case of the stacking gel (pH 6.8). A 30% acrylamide/bis-acrylamide ratio of 37.5:1, with a 2.7% crosslinker (Bio-Rad, Hercules, CA, USA), was used. Each sample (control, BSY, NSY, and TSY) was suspended in 4 × Laemmli sample buffer (Bio-Rad, Hercules, CA, USA) and treated under reducing conditions using β-mercaptoethanol, according to the manufacturer’s recommendations. After thermal treatment at 95 °C for 5 min in a water bath, followed by centrifugation for 3 min at 5.000 rpm, a volume of 20 µL of each sample was loaded into the wells. The electrophoresis was run at a constant voltage of 100 V for 100 min. The protein bands were fixed for 1 h by immersing the gels in a 40% methanol solution and 10% acetic acid, stained for 40 min in a 0.1% *w*/*v* Coomassie brilliant blue R-250 solution (Bio-Rad, Hercules, CA, USA) and de-stained for 30 min in 10% *v*/*v* acetic acid. Afterwards, the gel was maintained in MilliQ water for 24 h and photographed with a Canon PowerShot G16 digital camera (Canon Inc., Tokyo, Japan).

#### 3.3.4. Antioxidant Activity Assays

The antioxidant activity of the spent brewer’s yeast protein hydrolysates and of the peptide mixtures separated through ultrafiltration with membranes having cut-offs of 30 kDa, 10 kDa and 3 kDa was evaluated using the methods based on DPPH and ABTS^+^ radicals-scavenging activity, as detailed in Dumitrascu et al. [33]. The antioxidant activity was expressed as μM Trolox Equivalent (TE)/g dw yeast.

#### 3.3.5. Color Coordinates

The color coordinates were measured by using the Chroma Meter CR-410 (Konica Minolta Sensing Americas Inc., Ramsey, NJ, USA). The color coordinates were expressed considering the CIELab scale, where L* represents the luminosity (0—darkest black to 100—brightest white), a* represents the green (negative values)/red (positive values) colors and b* represents blue (negative values)/yellow (positive values) colors [5]. The measurements were performed on the yeast protein hydrolysates, after enzyme inactivation and prior to centrifugation.

#### 3.3.6. Foaming Properties

The foaming properties of the yeast protein hydrolysates were determined using the methods described by Liang et al. [28], with slight modifications. A volume (V_0_) of 50 mL protein solution of 6% (*w*/*v*) was subjected to foaming in a graduated cylinder, using the Ultra Turax^®^ IKA T18 basic homogenizer with the S18N-19G dispersing tool (IKA-Werke GmbH and Co. KG, Staufen, Germany). The volume of the foam generated after 2 min of homogenization at three different speed values of 5000, 7000 and 9000 rpm (V_f_) was used to calculate the foaming capacity (FC) as follows:

FC (%) = (V_f_ − V_0_)/V_0_ × 100 (4)

The foam collapse was observed over 30 min of storage at room temperature. The volume of the foam measured after 30 min (V_30_) was used to calculate the foam stability (FS) as follows:

FS (%) = (V_30_ − V_0_)/(V_f_ − V_0_) × 100 (5)

#### 3.3.7. Rheological Properties of the Emulsions

Emulsions were prepared by homogenizing for 5 min at a speed of 15,000× *g* (Ultra Turrax^®^ IKA T18, IKA-Werke GmbH and Co. KG, Staufen, Germany) the 1:1 (*v*/*v*) mixtures consisting of protein suspensions of 6% concentration (*w*/*v*) and sunflower oil (Spornic, Prutul SA, Galati, Romania).

The obtained emulsions were immediately used for rheological measurements at 20 °C, using a controlled-stress rheometer (AR2000ex, TA Instruments Ltd., New Castle, DE, USA) and a cone–plate geometry (cone angle of 2° and diameter of 40 mm). A closing gap of 1000 μm was selected for all measurements, and the edges were covered with mineral oil to avoid moisture loss.

The linear viscoelastic region (LVR) of the emulsions was first identified by running a strain sweep test in the oscillating strain domain of 0.1–100%, at a frequency of 1 Hz. The storage modulus (G′), loss modulus (G″) and complex modulus (G*) were further registered in the frequency domain of 0.1 to 100 Hz while running a frequency sweep test at constant strain within the LVR. The stepped flow was finally applied to measure the steady shear viscosities while raising the shear rate in the 0.1–100 s^−1^ domain. The results were analyzed by means of TA Rheology Advantage Data Analysis Software V 4.8.3. (TA Instruments, New Castle, DE, USA).

### 3.4. Statistical Analysis

The results are expressed as mean value followed by standard deviation. The significant differences between samples were assessed using one-way ANOVA, whereas post hoc analysis at *p* < 0.01 was performed with Tukey test for post hoc analysis. Correlations were determined by Pearson test. Minitab 19 (Minitab LLC, State College, PA, USA) software was employed to perform the statistical analysis tests.

## 4. Conclusions

The antioxidant properties and technological functionality of the yeast protein hydrolysates obtained using bromelain, neutrase and trypsin were investigated. When hydrolysis of the spent brewer’s yeast was carried out with neutrase, higher soluble solids and protein contents were released compared to the samples hydrolyzed with bromelain and trypsin. The luminosity of the hydrolysates was correlated with the soluble protein content. Regardless of the exogenous enzyme used for preparing the yeast protein hydrolysates, the maximum antioxidant activity assessed using the DPPH method was obtained after 43 h of hydrolysis. Regarding the peptides with a molecular weight lower than 3 kDa, the highest antioxidant activity was obtained in the case of the sample hydrolyzed with bromelain and the lowest for the samples prepared with neutrase. The technological functionality of the yeast proteins hydrolysates was estimated based on the foaming properties and rheological behavior of the emulsions. The results suggested that the exogenous enzyme-assisted hydrolysis is a promising approach for improving the foaming properties of the yeast proteins. The hydrolysates prepared with trypsin exhibited the best foaming properties. The rheological measurements indicated that the stability of the emulsions highly depends on the enzyme used for protein hydrolysis. Additional studies will be conducted to investigate in detail the bioactivity and cytotoxicity of the peptides obtained through enzymatic hydrolysis of the spent brewer’s yeast. Moreover, the functionality of the yeast proteins hydrolysates in complex matrices, such as the value-added food products, will be considered as well.

## Figures and Tables

**Table 1:** The solid residue content (g/100 g dw) of the extracts obtained from spent brewer’s yeast hydrolyzed with bromelain (BSY), neutrase (NSY) and trypsin (TSY) after different time intervals.

Sample	Hydrolysis Time (Hours)				
	4	8	21	43	67
Control	32.11 ± 2.96 ^aD^*	32.50 ± 1.81 ^aD^	34.74 ± 1.08 ^aC^	33.03 ± 0.23 ^aC^	32.47 ± 2.34 ^aC^
BSY	41.46 ± 1.71 ^bC^	39.44 ± 1.73 ^cC^	53.79 ± 5.51 ^aA^	52.58 ± 1.88 ^aB^	52.15 ± 1.33 ^aB^
NSY	55.80 ± 2.41 ^bA^	55.12 ± 3.75 ^bA^	60.69 ± 1.62 ^aA^	60.98 ± 1.74 ^aA^	62.18 ± 0.64 ^aA^
TSY	48.03 ± 2.46 ^aB^	48.33 ± 5.45 ^aB^	53.65 ± 0.54 ^aAB^	51.61 ± 2.34 ^aB^	50.43 ± 1.15 ^aB^

* Mean values that do not share the same lowercase letter (^a, b, c^) for the same row are statistically significant at *p* < 0.01, based on Tukey test. Mean values that do not share the same uppercase letter (^A, B, C, D^) for the same column are statistically significant at *p* < 0.01, based on Tukey test.

**Table 2:** The soluble protein content (g/100 g dw) of the extracts obtained from spent brewer’s yeast hydrolyzed with bromelain (BSY), neutrase (NSY) and trypsin (TSY) after different time intervals.

Sample	Hydrolysis Time (Hours)				
	4	8	21	43	67
Control	22.66 ± 1.62 ^bC^*	26.85 ± 0.00 ^abC^	24.38 ± 0.01 ^abD^	25.97 ± 4.94 ^abC^	34.31 ± 3.56 ^aC^
BSY	39.45 ± 2.52 ^cB^	43.07 ± 3.29 ^bcB^	51.09 ± 1.49 ^bC^	61.56 ± 0.27 ^aB^	67.46 ± 1.63 ^aB^
NSY	63.24 ± 1.13 ^cA^	68.97 ± 2.36 ^cA^	77.29 ± 0.85 ^bA^	85.51 ± 2.00 ^aA^	87.65 ± 0.00 ^aA^
TSY	41.52 ± 0.28 ^bB^	47.68 ± 1.08 ^bB^	57.82 ± 2.20 ^aB^	59.70 ± 2.08 ^aB^	59.84 ± 1.55 ^aB^

* Mean values that do not share the same lowercase letter (^a, b, c^) for the same row are statistically significant at *p* < 0.01, based on Tukey test. Mean values that do not share the same uppercase letter (^A, B, C, D^) for the same column are statistically significant at *p* < 0.01, based on Tukey test.

**Table 3:** The degree of hydrolysis (%) of the proteins from spent brewer’s yeast hydrolyzed with bromelain (BSY), neutrase (NSY) and trypsin (TSY) at different time intervals.

Sample	Hydrolysis Time (Hours)				
	4	8	21	43	67
Control	8.27 ± 0.69 ^aD^*	6.99 ± 0.24 ^bD^	6.30 ± 0.17 ^cD^	6.49 ± 1.04 ^bD^	7.92 ± 0.32 ^aD^
BSY	11.50 ± 0.97 ^cC^	9.76 ± 0.36 ^dC^	12.87 ± 0.86 ^cC^	16.79 ± 0.53 ^bB^	22.55 ± 1.16 ^aB^
NSY	22.70 ± 0.41 ^bA^	17.55 ± 0.21 ^cA^	26.73 ± 0.55 ^aA^	25.74 ± 0.98 ^aA^	26.91 ± 0.62 ^aA^
TSY	12.52 ± 0.46 ^dB^	12.00 ± 0.66 ^dB^	13.62 ± 0.16 ^cB^	14.31 ± 0.17 ^bC^	18.05 ± 0.66 ^aC^

* Mean values that do not share the same lowercase letter (^a, b, c, d^) for the same row are statistically significant at *p* < 0.01, based on Tukey test. Mean values that do not share the same uppercase letter (^A, B, C, D^) for the same column are statistically significant at *p* < 0.01, based on Tukey test.

**Table 4:** The ABTS^+^ and DPPH scavenging activity (μM TE/g dw) of the extracts obtained from spent brewer’s yeast hydrolyzed with bromelain (BSY), neutrase (NSY) and trypsin (TSY) at different time intervals.

Sample	Hydrolysis Time (Hours)				
	4	8	21	43	67
ABTS^+^ scavenging activity (μM TE/g dw)					
Control	757.6 ± 0.96 ^dC^*	774.5 ± 0.6 ^cD^	858.5 ± 1.92 ^bD^	1088 ± 119 ^aC^	1024 ± 55 ^aD^
BSY	840.6 ± 0.96 ^eB^	845.4 ± 2.3 ^dC^	1346 ± 28 ^cC^	1596 ± 21 ^bB^	1632 ± 2 ^aB^
NSY	857.7 ± 2.13 ^dA^	854.9 ± 1.9 ^dB^	1564 ± 6 ^cA^	1633 ± 7 ^bA^	1660 ± 4 ^aA^
TSY	855.6 ± 0.96 ^cA^	860.4 ± 4.1 ^cA^	1475 ± 34 ^bB^	1573 ± 8 ^aB^	1561 ± 44 ^aC^
DPPH scavenging activity (μM TE/g dw)					
Control	45.35 ± 3.17 ^dC^	26.43 ± 0.19 ^eD^	73.80 ± 0.19 ^bD^	83.89 ± 1.78 ^aD^	65.25 ± 1.8 ^cD^
BSY	45.21 ± 3.52 ^cC^	35.40 ± 1.38 ^dC^	88.10 ± 0.59 ^bC^	96.51 ± 2.57 ^aC^	88.38 ± 0.59 ^bC^
NSY	82.35 ± 1.98 ^cA^	59.9 ± 4.75 ^dA^	110.2 ± 0.59 ^bA^	116.9 ± 2.9 ^aA^	106.6 ± 4.9 ^bA^
TSY	66.37 ± 2.37 ^cB^	45.35 ± 5.15 ^dB^	94.13 ± 0.79 ^bB^	102.8 ± 2.7 ^aB^	95.25 ± 2.37 ^bB^

* Mean values that do not share the same lowercase letter (^a, b, c, d, e^) for the same row are statistically significant at *p* < 0.01, based on Tukey test. Mean values that do not share the same uppercase letter (^A, B, C, D^) for the same column are statistically significant at *p* < 0.01, based on Tukey test.

**Table 5:** The ABTS^+^ and DPPH scavenging activity of the extracts obtained from spent brewer’s yeast hydrolyzed for 67 h with bromelain (BSY), neutrase (NSY) and trypsin (TSY) and passed through ultrafiltration membranes with the cut-offs of 30, 10 and 3 kDa.

Sample	Membrane Cut-Off		
	30 kDa	10 kDa	3 kDa
ABTS^+^ scavenging activity (μM TE/g dw)			
Control	483 ± 7 ^bD^*	656 ± 109 ^aC^	511 ± 119 ^aD^
BSY	1223 ± 10 ^aA^	1208 ± 61 ^aA^	1126 ± 34 ^bB^
NSY	1203 ± 4 ^aB^	1286 ± 10 ^aA^	1139 ± 17 ^bA^
TSY	1086 ± 23 ^aC^	1088 ± 8 ^aB^	742 ± 42 ^bC^
DPPH scavenging activity (μM TE/g dw)			
Control	43.05 ± 2.98 ^aC^	40.25 ± 3.42 ^aD^	27.07 ± 1.75 ^bC^
BSY	60.15 ± 7.53 ^bB^	85.10 ± 5.30 ^aA^	50.06 ± 0.39 ^cA^
NSY	64.36 ± 1.57 ^bB^	68.00 ± 1.98 ^aB^	27.63 ± 0.30 ^cC^
TSY	71.08 ± 4.22 ^aA^	61.83 ± 2.21 ^bC^	36.60 ± 0.39 ^cB^

* Mean values that do not share the same lowercase letter (^a, b, c^) for the same row are statistically significant at *p* < 0.01, based on Tukey test. Mean values that do not share the same uppercase letter (^A, B, C, D^) for the same column are statistically significant at *p* < 0.01, based on Tukey test.

**Table 6:** The influence of the exogenous enzyme used for the spent brewer’s yeast hydrolysis (BSY—bromelain, NSY—neutrase, and TSY—trypsin) and hydrolysis time on the color parameters of the hydrolyzed yeast slurries.

Sample	Hydrolysis Time (Hours)				
	4	8	21	43	67
L*					
Control	79.97 ± 0.23 ^Aa^*	78.88 ± 0.14 ^cA^	80.01 ± 0.19 ^aA^	79.24 ± 0.16 ^bA^	79.38 ± 0.50 ^aA^
BSY	77.52 ± 0.20 ^Ab^	75.87 ± 0.10 ^bB^	74.66 ± 0.39 ^cB^	72.21 ± 0.46 ^dB^	69.83 ± 0.18 ^eC^
NSY	71.53 ± 0.26 ^Ad^	69.68 ± 0.11 ^bD^	66.88 ± 0.24 ^cD^	66.76 ± 0.29 ^cD^	66.01 ± 0.20 ^dD^
TSY	75.86 ± 0.33 ^aC^	73.34 ± 0.10 ^bC^	71.83 ± 0.37 ^cC^	71.15 ± 0.34 ^cC^	71.01 ± 0.27 ^dB^
a* coordinate					
Control	5.97 ± 0.05 ^dC^	5.99 ± 0.01 ^dD^	6.39 ± 0.03 ^cC^	7.16 ± 0.02 ^bB^	7.53 ± 0.01 ^aB^
BSY	6.6 ± 0.01 ^dB^	6.98 ± 0.02 ^cB^	6.62 ± 0.02 ^dB^	7.00 ± 0.03 ^bC^	7.22 ± 0.02 ^aC^
NSY	6.78 ± 0.03 ^eA^	7.36 ± 0.03 ^dA^	7.5 ± 0.03 ^cA^	7.53 ± 0.06 ^bA^	7.79 ± 0.02 ^aA^
TSY	5.83 ± 0.05 ^bD^	6.40 ± 0.02 ^aC^	5.42 ± 0.03 ^cD^	4.80 ± 0.03 ^eD^	4.98 ± 0.03 ^dD^
b* coordinate					
Control	12.93 ± 0.01 ^eC^	13.21 ± 0.01 ^dC^	13.91 ± 0.15 ^cC^	15.15 ± 0.00 ^bA^	16.29 ± 0.01 ^aA^
BSY	13.53 ± 0.06 ^dB^	13.46 ± 0.01 ^eB^	14.39 ± 0.08 ^cA^	14.57 ± 0.05 ^bB^	14.84 ± 0.04 ^aB^
NSY	14.38 ± 0.07 ^aA^	13.69 ± 0.02 ^cA^	14.24 ± 0.09 ^bB^	14.49 ± 0.09 ^aC^	14.38 ± 0.07 ^bC^
TSY	12.65 ± 0.04 ^bD^	12.94 ± 0.02 ^aD^	12.15 ± 0.01 ^cD^	12.1 ± 0.03 ^cD^	12.68 ± 0.02 ^bD^

* Mean values that do not share the same lowercase letter (^a, b, c, d, e^) for the same row are statistically significant at *p* < 0.01, based on Tukey test. Mean values that do not share the same uppercase letter (^A, B, C, D^) for the same column are statistically significant at *p* < 0.01, based on Tukey test.

**Table 7:** Foaming properties of the extracts obtained from spent brewer’s yeast hydrolyzed for 67 h with bromelain (BSY), neutrase (NSY) and trypsin (TSY).

Sample	Foaming Capacity, %			Foam Stability, %		
	5000 rpm	7000 rpm	9000 rpm	5000 rpm	7000 rpm	9000 rpm
Control	32.5 ± 3.5 ^cC^*	62.5 ± 3.5 ^dB^	92.5 ± 3.5 ^cA^	46.4 ± 5.1 ^cA^	58.0 ± 0.5 ^cA^	56.7 ± 1.7 ^dA^
BSY	75.0 ± 0.0 ^aC^	100.0 ± 0.0 ^bB^	122.5 ± 3.5 ^abA^	81.7 ± 2.4 ^abA^	86.3 ± 1.8 ^bA^	83.7 ± 0.5 ^bA^
NSY	55.0 ± 7.1 ^bC^	87.5 ± 3.5 ^cB^	120.0 ± 0.0 ^bA^	68.3 ± 2.4 ^bB^	81.4 ± 2.8 ^bA^	76.0 ± 1.5 ^cAB^
TSY	85.0 ± 0.0 ^aB^	125.0 ± 0.0 ^aA^	132.5 ± 3.5 ^aA^	85.3 ± 4.2 ^aA^	98.0 ± 2.8 ^cA^	94.4 ± 2.5 ^aA^

* Mean values that do not share the same lowercase letter (^a, b, c, d^) for the same row are statistically significant at *p* < 0.01, based on Tukey test. Mean values that do not share the same uppercase letter (^A, B, C^) for the same column are statistically significant at *p* < 0.01, based on Tukey test.

**Table 8:** Rheological properties of the emulsions prepared with extracts obtained from spent brewer’s yeast hydrolyzed for 67 h with bromelain (BSY), neutrase (NSY) and trypsin (TSY).

Sample	Stepped Flow Test		Strain Sweep Test	
	Viscosity (Pa·s) at Shear Rate of 1 s^−1^	Viscosity (Pa·s) at Shear Rate of 10 s^−1^	Critical Strain (γc), %	Yield Strain, (γy), %
Control	0.182 ± 0.002 ^a^*	0.025 ± 0.001 ^a^	0.36 ± 0.06 ^b^	3.17 ± 0.01 ^b^
BSY	0.178 ± 0.005 ^a^	0.023 ± 0.002 ^a^	1.43 ± 0.23 ^a^	25.22 ± 0.21 ^a^
NSY	0.109 ± 0.013 ^b^	0.032 ± 0.002 ^a^	0.36 ± 0.06 ^b^	1.00 ± 0.01 ^c^
TSY	0.105 ± 0.002 ^b^	0.022 ± 0.007 ^a^	0.32 ± 0.01 ^b^	-

* Mean values that do not share the same lowercase letter (^a, b, c^) for the same column are statistically significant at *p* < 0.01, based on Tukey test.

## Data Availability

Not applicable.
